# Beneficial Effects of Citrus Flavonoids on Cardiovascular and Metabolic Health

**DOI:** 10.1155/2019/5484138

**Published:** 2019-03-10

**Authors:** Ayman M. Mahmoud, Rene J. Hernández Bautista, Mansur A. Sandhu, Omnia E. Hussein

**Affiliations:** ^1^Physiology Division, Department of Zoology, Faculty of Science, Beni-Suef University, Egypt; ^2^Metropolitan Autonomous University, Laboratory of Bioenergetics and Cellular Aging, Department of Health Sciences, Division of Health and Biological Sciences, Mexico; ^3^Biomedical Sciences Department, Faculty of Veterinary & Animal Sciences, PMAS Arid Agriculture University, Pakistan

## Abstract

The prevalence of cardiovascular disease (CVD) is increasing over time. CVD is a comorbidity in diabetes and contributes to premature death. Citrus flavonoids possess several biological activities and have emerged as efficient therapeutics for the treatment of CVD. Citrus flavonoids scavenge free radicals, improve glucose tolerance and insulin sensitivity, modulate lipid metabolism and adipocyte differentiation, suppress inflammation and apoptosis, and improve endothelial dysfunction. The intake of citrus flavonoids has been associated with improved cardiovascular outcomes. Although citrus flavonoids exerted multiple beneficial effects, their mechanisms of action are not completely established. In this review, we summarized recent findings and advances in understanding the mechanisms underlying the protective effects of citrus flavonoids against oxidative stress, inflammation, diabetes, dyslipidemia, endothelial dysfunction, and atherosclerosis. Further studies and clinical trials to assess the efficacy and to explore the underlying mechanism(s) of action of citrus flavonoids are recommended.

## 1. Introduction

Diabetes mellitus (DM) is a metabolic disease characterized by chronic hyperglycemia and defective insulin secretion, insulin action or both [1, 2]. DM is associated with significant morbidity and mortality due to its related complications particularly on the cardiovascular system [3, 4]. Recent reports estimated that there are 415 million diabetic patients worldwide, and the number is projected to increase and may reach 642 million by 2040 [5]. The chronic and prolonged hyperglycemia in DM is associated with increased risk of developing cardiovascular disease (CVD) [6]. Hyperglycemia induces excessive generation of reactive oxygen species (ROS) in the diabetic heart, resulting in oxidative stress [7]. Hyperglycemia-mediated oxidative stress represents the main pathophysiological mechanism behind the development of diabetic cardiomyopathy (DCM) and many other cardiovascular alterations [8]. DCM is characterized by diastolic dysfunction, cardiac remodeling, hypertrophy, and altered cardiac energy metabolism [9, 10]. Within the diabetic heart, increased levels of ROS induce cardiac injury by direct damage of the cellular macromolecules, including lipids, proteins, and DNA [11, 12]. In addition to oxidative stress, hyperglycemia can induce mitochondrial dysfunction, inflammation, increased advanced glycation end products (AGEs), and activation of protein kinase C (PKC) and polyol pathways [10]. Moreover, dyslipidemia has emerged as a major factor in the pathogenesis of DCM [13].

Atherosclerosis is a chronic inflammatory process of large- and medium-sized arteries, characterized by the abnormal deposition of fibrous tissue, cholesterol, and lipid plaques in the inner most layer of the arteries [14]. This disease leads to narrowing of arteries and disturbs the basic structure of vessels which lead to partial and/or complete blockage of arteries. Atherosclerosis of coronary artery results in irregular blood flow which leads to ischemic heart failure and myocardial infarction [15]. Different risk factors are responsible for the pathogenesis of atherosclerosis, and those include hyperlipidemia, hypertension, endothelial dysfunction (ED), smoking, and diabetes. In addition, different inflammatory and immunological features play a pivotal role in the development of the disease process, as macrophages containing oxidizing particles discharge different inflammatory substances including cytokines and different growth factors such as intercellular adhesion molecule (ICAM-1); monocyte chemoattractant protein-1 (MCP-1); macrophage colony-stimulating factor; interleukin- (IL-) 1, 3, 6, 8, and 18; and tumor necrosis factor (TNF-*α*) [16, 17]. Cell proliferation and ROS production are accelerated by proinflammatory cytokines, which ultimately stimulate the metalloproteinases leading to expression of tissue factor, which results in leukocyte activation, ED, and initiation of atherosclerosis [18–20].

**Figure 1:**
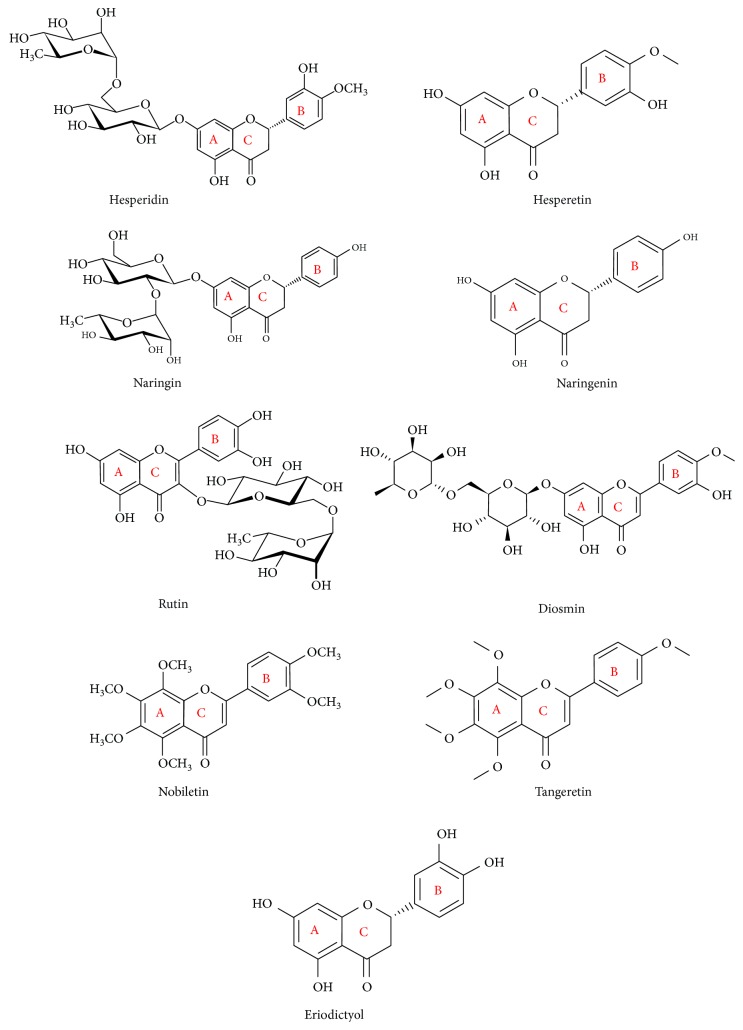
Chemical structure of the common citrus flavonoids.

Flavonoids are plant-based natural products that are very abundant and have multiple therapeutic benefits and biological activities. This diverse group of compounds exerts antihyperglycemic, antihyperlipidemic, anticarcinogenic, antihyperammonemia, nephroprotective, and hepatoprotective activities as we reported previously [21–29]. The basic structure of flavonoids involves 15-carbon atoms and 2 phenolic rings carrying one or more hydroxyl group (OH). According to their structure, flavonoids could be divided into 6 classes: flavanones, flavones, flavanols, isoflavones, flavonols, and anthocyanidins [30]. There are thousands of food flavonoid compounds existing in aglycone form or bound to glycosides [31, 32]. Dietary flavonoids in nature exist as glycosides, such as, glucoside, galactoside, arabinoside, rhamnoside, and rutinoside [33, 34]. All dietary flavonoids except flavanols are found in glycosylated forms [35], and deglycosylation is a critical step in the absorption and metabolism of flavonoid glycosides [36]. The flavonoid glycosides are water-soluble, whereas aglycones are more hydrophobic and can be easily absorbed [32, 37, 38]. Within the small intestine, only aglycones and some glucosides can be absorbed; however, flavonoids linked to a rhamnose moiety must be hydrolyzed by rhamnosidases of the microflora in the large intestine [39, 40]. The flavonoid glycosides are then absorbed, bound to albumin, and transported to the liver via the portal vein [41–43]. The intrahepatic metabolism of flavonoids is influenced by different factors [31], and flavonoids and their derivatives may undergo hydroxylation, methylation, and reduction [42]. Citrus fruits are notably rich in flavonoid compounds and represent an important source of dietary flavonoids, including hesperidin, hesperetin, naringin, naringenin, diosmin, quercetin, rutin, nobiletin, tangeretin, and others (Figure 1). These flavonoids are present in many citrus fruits, such as, bergamots, grapefruit, lemons, limes, mandarins, oranges, and pomelos [44]. The health-related effects of citrus flavonoids have been reported in several studies. Among their biological activities, citrus flavonoids possess radical scavenging, antioxidant, and anti-inflammatory properties. Given the role of oxidative stress in the pathogenesis of CVD, including DCM, and atherosclerosis, we aim in this review to focus on the mechanisms of action of citrus flavonoids in oxidative stress, diabetes, DCM, lipid metabolism, adipose tissue inflammation, ED, platelet function, and atherosclerosis.

## 2. Biological Activities of Citrus Flavonoids

### 2.1. Citrus Flavonoids and Oxidative Stress

Flavonoids possess multiple health benefits, including antioxidant and free radical scavenging, anti-inflammatory, and cytoprotective [45–48]. Given the role of oxidative stress and inflammation in the pathogenesis of obesity, diabetes, and CVD [4, 49–53], the antioxidant potential of flavonoids may play a key role in their beneficial therapeutic effects. The chemical structure of flavonoids indicates that they act as radical scavengers, oxygen quenchers, and hydrogen-donating antioxidants. Therefore, flavonoids can boost endogenous antioxidants and prevent the formation of ROS and their subsequent cell damage [54].

Flavonoids can prevent cell injury through the direct scavenging of free radicals and hence prevent their deleterious effects. Flavonoids are oxidized by free radicals, resulting in a more stable flavonoid radical and less reactive free radicals. Some flavonoids can directly scavenge superoxide, whereas others can scavenge peroxynitrite (ONOO•). The presence of OH groups permits high flavonoid reactivity against ROS and reactive nitrogen species (RNS). Flavonoids can stabilize OH•, peroxyl (ROO•), and ONOO• radicals. The antioxidant efficacy of a given flavonoid increases in function with the number of OHs in the structure of the molecule [48, 55]. For example, the 5-OH substitution and a 5,7-*m*-dihydroxy arrangement in the A-ring is an important feature of naringenin, making it a potent antioxidant with stabilized structure after donating H to the R^•^ [55].

Oxidative stress is a frequent pathological contributor to most liver diseases. The concerted work of oxidative stress and inflammation may increase production of the extracellular matrix (ECM) followed by fibrosis, cirrhosis, hepatocellular carcinoma (HCC), and finally liver failure [55]. Naringenin has been reported to suppress lipid peroxidation and protein carbonylation, enhance antioxidant defenses, scavenge ROS, modulate signaling pathways related to fatty acid metabolism, lower lipid accumulation in the liver, and thereby prevent fatty liver [55, 56].

By scavenging radicals, flavonoids can inhibit low-density lipoprotein (LDL) oxidation and therefore may have preventive action against atherosclerosis [57]. Several studies from Mahmoud's lab have documented the effect of flavonoids on cellular redox status and inflammation in different diseases, including HCC [29], diabetes [58], diabetic retinopathy [58], and drug-induced hepatotoxicity [28]. Other studies have demonstrated the beneficial role of citrus flavonoids in nonalcoholic fatty liver disease (NALFD), the most common liver disease caused by high fat consumption, vitamin and energy deficiency, and inflammatory processes [55, 56].

Citrus flavonoids have been shown to improve lipid metabolism, and their effects are thought to be mediated via their antioxidant capacity [59, 60]. Rutin is a powerful radical scavenger, and its scavenging ability may be due to its inhibitory activity on the enzyme xanthine oxidase (XO). The antioxidant effect of naringenin is primarily attributed to reducing ROS and enhancing the antioxidant defenses, including superoxide dismutase (SOD), catalase (CAT), and glutathione peroxidase (GPx) in chronic diseases [48].

Naringenin has shown a protective effect against nephrotoxicity induced by vancomycin, a drug used in severe infections. Vancomycin-induced rats treated with different doses of naringenin showed a significant amelioration in oxidative stress and apoptosis markers. Naringenin ameliorated serum creatinine and blood urea nitrogen levels and kidney nitric oxide (NO) and caspase-3/8 activities. However, the protective effect of naringenin was associated with the dose. At moderate doses, naringenin exerted a protective role, but at higher doses the protective effect was decreased [61]. *In vitro* treatment of the RAW264.7 cells with naringenin suppressed the inflammatory mediators and suppressed AGEs [62]. Rats treated with naringenin and quercetin for 14 days showed improved neurocognitive functions, enhanced antioxidant defenses, and suppressed lipid peroxidation in the brain [63]. In a rat model of diabetic retinopathy, naringenin attenuated oxidative stress and apoptosis and boosted the antioxidants. In addition, naringenin ameliorated the levels of brain-derived neurotrophic factor, tropomyosin-related kinase B, synaptophysin, B-cell lymphoma 2 (Bcl-2), Bcl-2-associated X protein (Bax), and caspase-3 in the retina of diabetic rats [64]. The beneficial therapeutic effects of naringenin are mediated, at least in part, via its antioxidant and radical scavenging properties [64]. However, the exact mechanisms underlying the antioxidant efficacy of naringenin are not fully understood. In the study of Wang et al., isolated neurons cultured *in vitro* under conditions of hypoxia and reoxygenation showed increased production of ROS. Treatment with naringenin resulted in a significant decrease in ROS production and improved mitochondrial function evidenced by increased levels of high-energy phosphates, increased mitochondrial membrane potential, and decreased apoptosis [65].

Hesperidin and its aglycone hesperetin, two flavonoids found primarily in oranges and lemons, have shown multiple beneficial effects, such as, anticarcinogenic, antihypertensive, antiviral, antioxidant, antidiabetic, hepatoprotective, and anti-inflammatory [28, 29, 58, 66]. Several studies have been conducted to explore the pharmacological activities, molecular targets, and mechanisms of action of hesperidin. Hesperidin can decrease capillary permeability, leakiness, and fragility [67, 68]. The antioxidant efficacy of hesperidin was not limited only to its radical scavenging activity, but it also enhanced the cellular antioxidant defenses via the extracellular signal–regulated kinase (ERK)/nuclear factor (erythroid-derived 2)-like 2 (Nrf2) signaling pathway [67]. Nrf2 is a redox-sensitive transcription factor that activates the transcription of antioxidant and cytoprotective enzymes [4]. Recently, studies have focused on the protective effects of hesperidin and hesperetin against ROS and oxidative stress. In this context, we have previously demonstrated the antioxidant activity of hesperidin against hyperglycemia-induced oxidative stress in high-fat diet (HFD)/streptozotocin- (STZ-) induced diabetic rats. Hesperidin significantly decreased lipid peroxidation and increased the levels of reduced glutathione (GSH), vitamin C, and vitamin E and enhanced the activity of antioxidant enzymes SOD, CAT, and GPx in type 2 diabetic rats [58]. We also demonstrated the antioxidant efficacy of hesperidin in a rat model of cyclophosphamide-induced liver injury. Our results showed suppressed lipid peroxidation, NO, inducible nitric oxide synthase (iNOS), and nuclear factor-kappaB (NF-*κ*B) and boosted enzymatic and nonenzymatic antioxidant defenses in the liver of hesperidin-treated rats. We reported that the upregulation of peroxisome proliferator-activated receptor (PPAR*γ*) mediated, at least in part, the antioxidant and anti-inflammatory potential of hesperidin [28]. More recently, we investigated the antioxidant efficacy of hesperidin in a hepatocarcinogenesis rat model. Our results showed the ability of hesperidin to prevent the increased production of ROS, NO, and lipid peroxides and to enhance both enzymatic and nonenzymatic defenses in the liver of rats subjected to chemically induced hepatocarcinogenesis. In addition, we reported that the mechanism of action of hesperidin included upregulation of PPAR*γ* and Nrf2/antioxidant response element (ARE)/antioxidant signaling pathways [29].

In addition to its ability to upregulate PPAR*γ* and Nrf2 signaling pathways, there is evidence that attenuation of endoplasmic reticulum (ER) stress is one of the effects of hesperidin. In this context, treatment of the ovarian cancer cell line A2780 with hesperidin decreased the viability in a dose- and time-dependent manner. This effect was mediated via induction of apoptosis as shown by the increased levels of cleaved caspase-3. Hesperidin upregulated the protein expression levels of anti-CCAAT/enhancer-binding protein- (C/EBP-) homologous protein/growth arrest and DNA damage-inducible gene 153 (GADD153), glucose-regulated protein (GRP) 78, and cytochrome *c*. These findings point to the role of ER stress signaling in mediating the impact of hesperidin on A2780 cells [69].

A recent study conducted by Wunpathe et al. [70] demonstrated the role of hesperidin in suppressing excessive ROS production mediated via renin-angiotensin system-mediated NADPH oxidase (NOX2) overexpression in hypertensive rats. In a rat model of two-kidney-one-clipped (2K-1C) hypertension, hesperidin reduced blood pressure in a dose-dependent manner and decreased plasma angiotensin (AT) II levels and aortic AT I receptor protein expression. In addition, hesperidin attenuated oxidative stress via suppressing NADPH oxidase in hypertensive rats [70].

The free radical-scavenging and immunomodulatory properties of hesperidin have been postulated to mediate its protective effect against X-irradiation-induced oxidative damage. Exposure to X-irradiation induced cardiovascular complications, including myocardial degeneration, vascular leakage, myocyte necrosis, development of plaque, inflammation, and fibrosis. Rats receiving hesperidin showed decreased cardiac lipid peroxidation, inflammation, fibrosis, and other complications and enhanced the activity of antioxidant enzymes [71].

Hesperetin, the aglycone of hesperidin, possesses a well-documented antioxidant efficacy. In a rat model of lead acetate-induced oxidative stress, hesperetin showed a significant antioxidant efficacy evidenced by the decreased lipid peroxidation and increased levels of GSH and activity of SOD, CAT, and GPx [68]. Hesperetin exerted a protective effect against oxidative stress in the testis of diabetic rats. Oral administration of hesperetin for 45 days suppressed ROS production, protein carbonylation, and oxidative DNA damage. In addition, hesperetin ameliorated GSH, SOD, CAT, and GPx in the testicular tissue of diabetic rats. In conjunction with attenuating oxidative stress, hesperetin prevented inflammation and apoptosis as evidenced by the low levels of proinflammatory cytokines and caspase-3 activity in diabetic rats [72]. Furthermore, hesperetin exerted a cardioprotective effect in doxorubicin-induced rats. Administration of hesperetin for 5 weeks reduced cardiac lipid peroxidation, increased GHS levels, and prevented oxidative DNA damage and apoptosis as shown by comet and terminal deoxynucleotidyl transferase-mediated dUTP nick-end labeling (TUNEL) assays, respectively [73].

The antioxidant capacity of other citrus flavonoids, including nobiletin, rutin, and tangeretin, has also been tested. Nobiletin, tangeretin, 5-demethylnobiletin (5-DN), and 5-demethyltangeretin (5-DT) are polymethoxyflavones found in aged citrus peels. These flavonoids have been reported to ameliorate cell tolerance, ROS production, and lipid peroxidation in *Saccharomyces cerevisiae* [74]. In mutant *Saccharomyces cerevisiae* deficient in glutathione synthase, CAT, or SOD, nobiletin, tangeretin, 5-DN, and 5-DT activated CAT under stress induced by carbon tetrachloride (CCl_4_), hydrogen peroxide (H_2_O_2_), and cadmium sulfate [74]. Nobiletin has also protected human retinal pigment epithelial cells against damage induced by H_2_O_2_ as shown by increased cell viability and suppressed ROS and activity of caspases [75]. The protective effect of nobiletin was associated with increased phosphorylation of protein kinase B (PKB/Akt), pointing to the role of phosphoinositide 3-kinase (PI3K)/Akt signaling in mediating the effects of nobiletin [75]. Through its ability to prevent excessive production of ROS, nobiletin inhibited cadmium-induced neuronal apoptosis and modulated c-Jun N-terminal kinase (JNK)/ERK1/2 and Akt/mTOR signaling, expression of the kinases MKK and ASK1, and phosphorylation of S6K1, Akt, and 4E-BP1 [76]. Previous research from our lab has demonstrated the antioxidant efficacy of rutin. In a rat model of hyperammonemia, rutin prevented lipid peroxidation and improved the antioxidant defenses of GSH, SOD, and GPx [26]. In type 2 diabetic rats, rutin inhibited hyperglycemia-induced oxidative stress and increased the hepatic antioxidant defenses [22]. Furthermore, rutin protected against oxidative stress in a rat model of hepatocarcinogenesis [77].

### 2.2. Citrus Flavonoids and Lipid Metabolism

Lipids are essential for maintaining various physiologic and homeostatic processes within the body. Dysregulation of the lipid and lipoprotein metabolism is one of the major risk factors leading to CVD, obesity, diabetes, and inflammation [45, 46, 59]. Several studies have demonstrated the beneficial role of citrus flavonoids in modulating lipid metabolism and attenuating several diseases, including obesity and atherosclerosis. However, the mechanisms underlying the therapeutic effects of citrus flavonoids are not fully understood. While human studies have emphasized the dose, bioavailability, efficacy, and safety, citrus flavonoids suppressed atherogenesis through ameliorating metabolic parameters and their direct impact on the vessel wall in rodents [59]. Citrus flavonoids can control calorie intake versus expenditure and regulate lipid metabolism, and their use as safe and natural alternatives to treat obesity is currently under investigation.

Although hesperidin and naringin reduced serum total and LDL-cholesterol in rodent models of diabetes [21], human studies showed no effect on serum cholesterol levels in moderately hypercholesterolemic men and women [78]. *In vitro* treatment of the hepatoma cell line HepG2 with naringenin and hesperetin for 4 hr reduced apoB100 accumulation in the media [79]. Naringenin and hesperetin suppressed microsomal triglyceride transfer protein and acyl-CoA:cholesterol acyltransferase in HepG2 cells [80]. Other *in vitro* studies using HepG2 cells showed inhibited apoB secretion and cholesterol synthesis following treatment with tangeretin and nobiletin, whereas sinesetin, hesperetin, and naringenin exerted weak effects [81]. The different effects of citrus flavonoids on apoB secretion and cholesterol synthesis could be attributed to differences in their molecular structure [81, 82].

The sterol regulatory element-binding proteins (SREBPs), transcriptional regulators of lipid synthetic genes, have been assumed to be implicated in mediating the effects of citrus flavonoids on lipid metabolism. In this context, a mutation of the SRE in the LDL receptor (LDLR) gene upstream region attenuated the effects of hesperetin and nobiletin in HepG2 cells [81]. Hesperidin stimulated LDLR gene expression in HepG2 cells via increasing the phosphorylation of PI3K and ERK1/2 and SREBP-2 mRNA abundance [83]. These effects can reduce plasma LDL levels and hence show the cardioprotective potential of hesperidin [83]. HepG2 cells with luciferase reporter-gene constructs incorporating the promoters of SREBP-1a, -1c, and -2, and LDLR, treated with 200 *μ*M naringenin in lipoprotein-deficient medium (LPDM), showed increased SREBP-1a promoter activity after 4 hr. After treatment for 24 hr, the gene expression levels of SREBP-1a, -1c, and -2 and LDLR promoter-constructs were increased [84]. In addition, naringenin suppressed SREBP-1c acetyl-CoA carboxylase and fatty acid synthase mRNA expression in HepG2 cells [84].

Other mechanisms mediating the effects of citrus flavonoids on lipid metabolism have been postulated. Hesperidin might be implicated in ghrelin secretion from stomach. Ghrelin may be related with the pathophysiological mechanisms of a variety of human disorders, including lipodystrophies [85]. Using *Caenorhabditis elegans* as a model, Peng et al. showed that hesperidin decreased fat accumulation; downregulated the expression of stearoyl-CoA desaturase, fat-6, and fat-7; and suppressed other genes involved in lipid metabolism, including pod-2, mdt-15, acs-2, and kat-1 [86]. In addition, mutations of fat-6 and fat-7 reversed fat accumulation inhibited by hesperidin [86].

Millar et al. have recently discussed the effects of flavonoids on reverse cholesterol transport (RCT), high-density lipoprotein (HDL) metabolism, and HLD function [46]. Given the role of inflammation in the induction of dysfunction of HDL particles, flavonoids can improve HDL function via attenuating oxidative stress and inflammation [46]. Previous work from our laboratory showed improved serum HDL levels in type 2 diabetic rats treated with hesperidin and naringin [21]. Long-term consumption of flavonoid-rich foods has been associated with improved circulating levels of total cholesterol, triglycerides, and LDL-cholesterol [87]. Preclinical *in vitro* and *in vivo* studies reported the influence of flavonoids on RCT and HDL function by regulating the activity and expression of hepatic paraoxonase 1 and cholesterol efflux from macrophages [46]. However, clinical studies targeting the effect of citrus flavonoids on HDL function are lacking [46].

Studies on the effects of flavonoids such as apigetrin (apigenin 7-*O*-glucoside) on adipogenesis have suggested similar effects for citrus flavonoids. Apigetrin, a flavonoid present in several plant leaves and seeds, significantly inhibited lipid accumulation and reduced the gene expression levels of C/EBP-*α*, PPAR-*γ*, SERBP-1c, fatty acid synthase (FAS), and proinflammatory cytokines in 3T3-L1 cells [88]. Similar effects have been exerted by citrus flavonoids. In 3T3-L1 adipocytes, nobiletin, an *O*-methylated flavone isolated from citrus peels, upregulated the beige-specific genes Cd137, Cidea, Tbx1, and Tmem26 and the protein expression of PKA and p-AMPK (5′-adenosine monophosphate-activated protein kinase) [89]. In addition, nobiletin upregulated the key transcription factors responsible for remodeling of white adipocytes, induced mitochondrial biogenesis, modulated several proteins related to lipid metabolism (CPT1, ACOX1, FAS, SREBP, SIRT1, and p-PLIN), and suppressed JNK and c-Jun [89]. Therefore, the citrus flavonoid nobiletin can induce browning and ameliorate stress in white adipocytes [89]. The effects of pure total flavonoids on lipid metabolism have also been tested. HFD-fed rats treated with the pure total flavonoids from *Citrus aurantium* for 4 weeks showed improved body weight, ameliorated serum cholesterol and triglycerides, enhanced antioxidant defenses, and upregulated gene and protein expression levels of PPAR-*α* and LPL [90].

### 2.3. Citrus Flavonoids and Adipose Tissue Inflammation

Adipose tissue stores lipid in the form of triglycerides and secretes a variety of mediators that regulate a number of cellular processes. It secretes a variety of adipocytokines and is therefore currently considered an endocrine organ. In addition to the rapid expansion of adipose tissue [59], chronic low-grade inflammation associated with insulin resistance and other metabolic disturbances are characteristic features of obesity [59, 91–93].

Flavonoids possess a potent anti-inflammatory potential, and several studies demonstrated their ability to attenuate inflammation associated with different diseases [25–29, 58, 59, 94]. Citrus fruits represent a source of flavonoids, and their consumption has been associated with reduced cardiovascular events that can also be associated with obesity, suggesting their cardioprotective potential [92, 95]. Multiple *in vitro* and *in vivo* studies provided strong evidence supporting the protective effect of flavonoids against vascular disturbances associated with obesity [92, 93]. The anti-inflammatory potential of flavonoids may be attributed to their ability to bind cyclooxygenases (COXs). COXs catalyze the conversion of arachidonic acid into prostaglandins and thromboxanes. COX-2 is an inducible form that is expressed upon stimulation and produces prostaglandins for the induction of the inflammation and pain [57]. *In silico* studies have demonstrated the ability of flavonols, flavones, and flavanones to bind COX-2, and this can help in developing potent inhibitors for the treatment of inflammation [57].

In lipopolysaccharide- (LPS-) stimulated RAW 264.7 cells, narangenin suppressed TNF-*α* and IL-6 release in a dose-dependent manner. Narangenin treatment downregulated gene expression levels of COX-2, TNF- *α*, IL-6, iNOS, and NOX-2 in LPS-stimulated macrophages [96]. These findings demonstrate the potent anti-inflammatory potential of narangenin. Other flavonoids, including apigenin, genistein, and kaempferol, have exerted COX-2 inhibitory effects via suppressing NF-*κ*B activation. Oroxylin A (5,7-dihydroxyflavone 6 methyl ether), a flavone isolated from *Scutellaria radix*, showed a similar effect where it suppressed iNOS and COX-2 through inhibition of NF-*κ*B activation [97]. In addition, pure flavonoids and flavonoid-enriched extracts can reduce the expression of cytokines and COX-2 [98].

Previous work from our lab has shown the anti-inflammatory effect of hesperidin and naringin in HFD/STZ-induced diabetic rats. Both flavonoid compounds decreased the levels of circulating proinflammatory cytokines and downregulated the expression of IL-6 in adipose tissue [27, 58]. A recent study by Ke et al. [99] showed that naringenin reduced adipose tissue mass, adipocyte size, and body weight and ameliorated adipose tissue inflammation in HFD-fed obese ovariectomized mice [99]. The same group demonstrated that naringenin decreases adipose tissue mass and attenuates metabolic disturbances in ovariectomized mice. Naringenin-fed ovariectomized mice exhibited over 50% reduction in subcutaneous and visceral adiposity, decreased hepatic lipid accumulation, and significantly downregulated MCP-1 and IL-6 mRNA in perigonadal adipose tissue [100].

Hesperetin and naringenin showed anti-inflammatory potential in mouse adipocytes. Both flavonoid compounds inhibited TNF-*α*-stimulated free fatty acid (FFA) release and blocked the activation of NF-*κ*B and ERK pathways in mouse adipocytes. Through ERK signaling inhibition, hesperetin and naringenin prevented the suppressing effect of TNF-*α* on the antilipolytic genes perilipin and PDE3B. In addition, the suppressive effect of hesperetin and naringenin on NF-*κ*B resulted in IL-6 downregulation and subsequently reduced FFA secretion from mouse adipocytes [101]. During the differentiation of adipocytes, naringenin has been reported to inhibit toll-like receptor- (TLR-) 2 expression, an effect mediated via upregulation of PPAR*γ* [102]. In this context, hesperidin has been reported to activate PPAR*γ* signaling in hepatocytes [28, 29]. In differentiated adipocytes, naringenin inhibited TNF-*α*-induced activation of TLR2 and NF-*κ*B [102]. *In vivo* studies showed that naringenin suppresses the infiltration of macrophages into the adipose tissue of mice fed a HFD for 14 days [103]. In addition, naringenin inhibited the c-Jun NH2-terminal kinase pathway and subsequently downregulated MCP-1 expression in the adipose tissue of HFD-fed mice [103]. *In vitro* cultured/cocultured adipocytes and macrophages showed suppressed MCP-1 expression following treatment with naringenin [103].

The beneficial role of naringenin and nobiletin in obesity and adipose tissue has been supported by using knockout mice. In the study of Burke et al. [92], *Ldlr*^−/−^ mice fed a high-fat-high cholesterol diet and treated with naringenin and nobiletin exhibited a significant improvement in metabolism and decreased obesity.

## 3. Therapeutic Potential of Citrus Flavonoids in Diabetes and DCM

### 3.1. Citrus Flavonoids and DM

DM occurs as a consequence of irregular catabolism and anabolism of carbohydrates, lipids, and proteins because of insulin resistance or hypoinsulinism [104]. On the basis of etiology and clinical signs, DM is classified into three types, type 1 DM, type 2 DM, and gestational DM. Type 1 DM is an insulin-dependent or juvenile diabetes and also termed as diabetes insipidus. Clinically, it is characterized by an autoimmune disorder against *β*-cells present in the islets of Langerhans of the endocrine pancreas. Around 5-10% of all diabetic patients are suffering from type 1 DM [105]. The initial incidence of type 1 DM usually occurs at the age of 4 years, or when the individual reaches in early adolescence and puberty, i.e., below the age of 20 years. In the early stages, individuals suffering from type 1 DM show mild fasting hyperglycemia, and this may progress to severe hyperglycemia and/or ketoacidosis, indicating impaired function of pancreatic *β*-cells. Upon diagnosis, 80-90% of patients suffering from type 1 DM will have elevated levels of auto-antibodies to insulin, including glutamic acid decarboxylase (GAD65), and tyrosine phosphates IA-2 and IA-2ß [106]. Type 1 DM signs are extreme urination and thirst, episodic hunger, gradual weight, and vision loss [106].

Type 2 DM is a non-insulin-dependent or adult onset of diabetes. Currently, type 2 DM is the most prevalent type of diabetes in the world and accounts for 90-95% patients [107]. Type 2 DM is considered as heterogeneous disease, because multiple factors are mixed up in its progression, including obesity, lack of physical activity, hypertension, and dyslipidemia. In this type of diabetes, the body produces sufficient amount of insulin but due to cellular resistance, it remains ineffective. Upon diagnosis of type 2 DM, almost every patient has some degree of impaired insulin secretion [108].

Gestational diabetes mellitus (GDM) is present or diagnosed during the 2nd/3rd trimester of pregnancy. Although GDM is a tentative disorder, it may increase the chances of getting type 2 DM later in life. Women with elevated level of blood glucose during pregnancy are diagnosed with GDM. Normally, GDM starts during the 24th week of pregnancy. Oral glucose tolerance test (OGTT) is recommended in high-risk women for the diagnosis of GDM. Women diagnosed with GDM are in jeopardy of elevated blood pressure, fetal macrosomia, and difficulty in vaginal birth [109]. Although GDM disappears after pregnancy, it may reappear in future pregnancies and may lead to type 2 DM in later stages of life. Additionally, the infants of GDM mothers are at threat of type 2 DM development during adolescence or in early adulthood [109].

Antioxidants are compounds which have the ability to delay or inhibit the oxidation of different molecules in the body. Although a low amount of ROS is beneficial in cell signaling, increased ROS is the major cause of cell death [110]. Different studies have proposed that phytochemicals either from fruit or vegetable sources can protect cells from ROS-induced damage [111].

Rutin, a natural citrus flavonoid found in fruits and vegetables, has effective efficacy in lowering hyperglycemia and also acts as an antioxidant [112]. A previous trial has shown that rutin supplementation significantly decreases glucose levels in diabetic patients [113]. Two studies have documented protective effects of rutin in diabetic rodent models [22, 114]. Another study showed that chronic hyperglycemia and dyslipidemia are a potential source of ROS in diabetes and may be a source of oxidative stress through different mechanisms, including autoxidation of glucose, lipid peroxidation, the polyol pathway, and glycosylation [115]. *In vivo*, rutin suppressed oxidative stress and partly reduced hyperglycemia and dyslipidemia in healthy rats but produced significant reduction in blood glucose and increased the activity of carbohydrate metabolic enzymes in diabetic rats [112]. Rutin increased insulin levels by stimulating the intact *ß* cells to produce insulin and may protect functional *ß* cells from further damage [112]. Previous research from our laboratory showed that the antidiabetic effect of rutin is mediated via ameliorating hyperglycemia, hyperlipidemia, insulin secretion, oxidative stress, inflammation, gluconeogenesis, glycogenolysis, peripheral glucose uptake, and intestinal glucose absorption in type 2 diabetic rats [22].

Nobiletin is another citrus flavonoid possessing adipocyte differentiation inhibitory activity [116] and can reduce the development of obesity which is directly correlated with type 2 diabetes. Nobiletin can act as an antidiabetic agent [117] and interferes with the differentiation of the 3T3-L1 preadipocyte cell line by inhibiting the extracellular signaling-regulated protein kinase signal pathway [116]. A study conducted by Lee et al. [117] showed that nobiletin has significant effects, including enhancement of Akt phosphorylation and glucose transporter- (GLUT-) 1 expression in complete cellular lysates and GLUT-4 in plasma membranes of white adipose tissue and muscles. In the same study, the effects of nobiletin were evaluated on the metabolism of glucose and insulin sensitivity in obese and diabetic *ob/ob* mice, where results have shown that 5-week treatment with nobiletin improved the circulating glucose level, homeostasis model assessment (HOMA) index, and results of OGTT.

Diosmin (DS) is a common component of many citrus fruits and has an ability to stimulate the activity of ß cells [118, 119]. A previous study has shown that oral treatment with DS for 45 days in diabetic rats significantly reduced plasma glucose level and enhanced the activity of hexokinase and glucose-6-phosphate dehydrogenase (G6PD) [118].

Hesperidin and naringin are very common citrus flavonoids and not only attenuate the diabetic condition but also can revoke neuropathic pain by controlling hyperglycemia and hyperlipidemia which upregulate the generation of free radicals and release of proinflammatory cytokines [120]. We have conducted different *in vivo* and *in vitro* studies to explore the mechanisms underlying the antidiabetic effects of hesperidin and naringin. In one study, both hesperidin and naringin attenuated hyperglycemia-induced oxidative stress and inflammation in HFD/STZ-induced type 2 diabetic rats. Both compounds reduced hyperglycemia, glycosylated hemoglobin levels, lipid peroxidation, TNF-*α*, and IL-6 and enhanced enzymatic and nonenzymatic antioxidant defenses [58]. In another study, both hesperidin and naringin prevented hematological alterations and modulated the expression of IL-6 and adiponectin in the adipose tissue of type 2 diabetic rats [27]. We have also shown that hesperidin and naringin improved serum insulin, hepatic and muscle glycogen, and gene and protein expression of GLUT-4. In addition, both compounds ameliorated hepatic glucose output, peripheral glucose uptake, intestinal glucose absorption, and glucose-stimulated insulin secretion from isolated islets of Langerhans [121].

Eriodictyol is a lemon citrus flavonoid and has significant ability to reduce oxidative stress in diabetic rats. It reduces the retinal vascular endothelial growth factor (VEGF), TNF-*α*, ICAM-1, and NO production, and it also has potential to downregulate diabetes-related lipid peroxidation [122]. Eriodictyol treatment may upregulate mRNA expression of PPAR*γ*2 and lipocyte-specific fatty acid-binding protein and the protein level of PPAR*γ*2 in differentiated 3T3-L1 adipocytes. In addition to these effects, eriodictyol also reactivated Akt in HepG2 cells with high glucose- (HG-) induced insulin resistance [123]. Insulin resistance has a close affinity with irregular signaling through IRS-1, P13k, and Akt pathways [124].

### 3.2. Citrus Flavonoids and DCM

**Figure 2:**
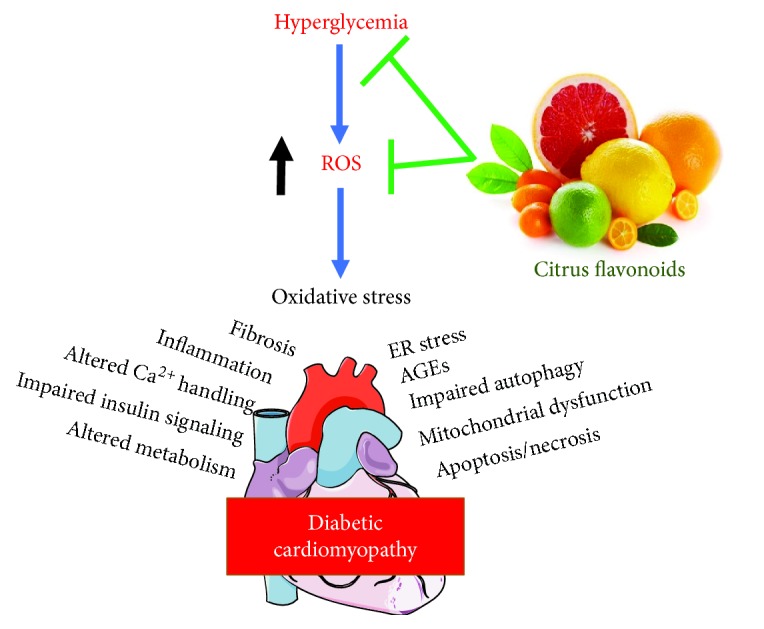
Citrus flavonoids protect against hyperglycemia-induced ROS in the diabetic heart.

DM is associated with increased risk of developing CVD, the principal cause of death and disability in people with diabetes [6]. DCM describes DM-associated pathological changes in the myocardium, independent of ischemic heart disease or hypertension. The prevalence of DCM has remarkably increased over the past decades [125] and is characterized by diastolic dysfunction, cardiac remodeling, hypertrophy, and altered cardiac energy metabolism [9, 10]. Hyperglycemia-induced excessive production of ROS is the main underlying mechanism of diabetes-induced cardiomyocyte damage [126]. Prolonged hyperglycemia can induce metabolic and molecular changes leading to myocardial injury [127]. Redox imbalance in the diabetic heart leads to oxidative DNA damage and accelerated myocardial apoptosis [128]. Other mechanisms involved in DCM include mitochondrial dysfunction, inflammation, increased AGEs, activation of PKC, and increased flux of hexosamine and polyol pathways [10] (Figure 2). Mitochondrial dysfunction plays a crucial role in the development and progression of DCM [129]. Hyperglycemia impairs the function of mitochondria by altering mitochondrial Ca^2+^ handling, energy metabolism and oxidative phosphorylation, dynamics, and biogenesis [130]. Hyperglycemia provokes nonenzymatic reaction of glucose with protein amino groups or lipids, leading to increased formation of AGEs [131]. Within the myocardium, AGE accumulation induces structural changes in several proteins, as well as Ca^2+^ handling, and consequently leads to myocardial stiffness [132]. In addition, AGE accumulation can provoke myocardial fibrosis by increasing collagen cross-linkage, impaired cardiac relaxation, and diastolic dysfunction [133]. Hyperglycemia can also activate the hexosamine biosynthetic pathway and increase *N*-acetylglucosamine (GlcNAc), leading to more ROS generation. Increased levels of GlcNAc may induce deactivation of antioxidant defense enzymes via O-GlcNAcylation [134]. Moreover, dyslipidemia, which includes lipoprotein abnormalities, has emerged as a major factor in the pathogenesis of DM-associated CVD [13]. Recently, we reported elevated serum lipids associated with a pronounced increase in cardiovascular risk indices in STZ-induced diabetic rats [135]. Dyslipidemia in diabetic rats has been associated with oxidative stress, inflammation, myocardial fibrosis, and multiple histopathological alterations [135].

Given the role of hyperglycemia, dyslipidemia, and oxidative stress in the pathogenesis of DCM, citrus flavonoids can attenuate myocardial damage in DM via antihyperglycemic, antihyperlipidemic, and antioxidant potential. In this context, several studies have reported the beneficial therapeutic effects of citrus flavonoids in diabetic cardiovascular complications. Hesperidin has been demonstrated to exert a cardioprotective effect in ischemic heart disease in diabetic rats [136]. Hesperidin activated PPAR*γ* signaling and reduced left ventricular end-diastolic pressure and mean arterial pressure in diabetic rats [136]. The efficacy of hesperidin to upregulate PPAR*γ* signaling has been supported by our recent study showing activated hepatic PPAR*γ* following hesperidin supplementation in cyclophosphamide-induced rats [28] and in an experimental model of hepatocarcinogenesis [29]. Hesperetin, the aglycone of hesperidin, has been recently reported to inhibit inflammation and fibrosis in the heart of STZ-induced diabetic rats by suppressing the NF-*κ*B signaling pathway [137]. Treatment of diabetic rats with hesperetin downregulated the expression of proinflammatory cytokines, adhesion molecules, and collagen I and III; inhibited NF-*κ*B activation; and decreased collagen deposition in the heart [137].

Naringin protected cardiomyocytes against hyperglycemia-induced injury both *in vitro* and *in vivo* as reported by You et al. [138]. Pretreatment of cardiomyocytes with naringin prevented high glucose-induced oxidative stress, apoptosis, and increased mitochondrial membrane potential (MMP) and NF-*κ*B p65 phosphorylation [138]. These findings were confirmed by *in vivo* treatment of STZ-induced diabetic rats with naringin. Diabetic rat hearts treated with naringin showed increased expression of ATP-sensitive K^+^ channels and SOD and decreased the ADP/ATP ratio and NOX4 expression [138]. Recently, Zhang et al. [139] showed the involvement of oxidative stress and ER stress in DCM and the ameliorative role of naringin. STZ-induced diabetic rats treated with naringin for 8 weeks exhibited improved glucose tolerance; enhanced cardiac antioxidants; decreased cardiac lipid peroxidation; downregulated mRNA and protein expression levels of GRP78, CHOP, and caspase-12; and improved the histological appearance of the myocardium [139]. These findings point to the role of naringin in ameliorating mitochondrial ROS production and inhibiting the ER stress-mediated apoptosis [139]. The aglycone form of naringin, naringenin, showed cardioprotective effects in STZ-diabetic mouse heart. Naringenin ameliorated cardiac hypertrophy in HFD/STZ-diabetic mice through upregulating both the gene and protein expression of PPARs, CYP2J3, and 14,15-EET [140].

In STZ-induced male diabetic mice, nobiletin attenuated oxidative stress, inflammation, and cardiac dysfunction as reported by Zhang et al. [141]. Echocardiography and hemodynamic measurements revealed improved cardiac function in diabetic mice treated with nobiletin. The cardioprotective mechanism of nobiletin included the suppression of NADPH oxidase-mediated ROS production and downregulated the expression of transforming growth factor- (TGF-) *β*1, fibronectin, collagen, JNK, P38, and NF-*κ*B. Therefore, nobiletin was able to inhibit NF-*κ*B activation and mitigate fibrosis in the diabetic mouse heart [141].

## 4. Therapeutic Potential of Citrus Flavonoids in Endothelial Dysfunction (ED) and Atherosclerosis

### 4.1. Citrus Flavonoids and ED

**Figure 3:**
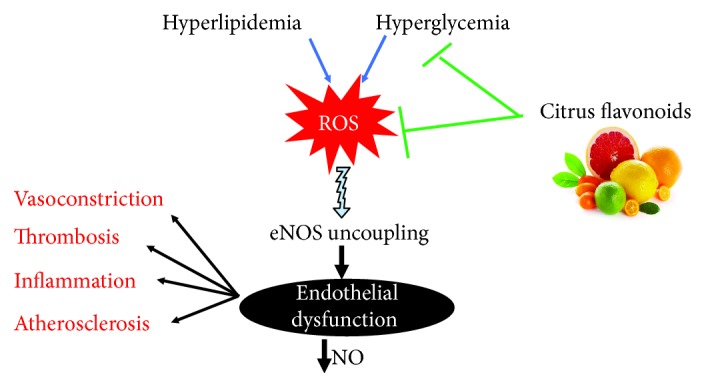
Citrus flavonoids prevent eNOS uncoupling and decreased NO production via their antioxidant activity.

Endothelial cells produce different and important vasoactive substances for the regulation of the proper vascular function and maintenance of vascular tone in the body. These substances are endothelium-derived hyperpolarizing factor (EDHF), NO, carbon monoxide, prostacyclin, endothelin, vasoactive prostanoids, and superoxide [142]. ED is a complex disease, and several factors are responsible for its initiation. ED is characterized by reduced bioavailability of NO because of eNOS uncoupling which might be a consequence of oxidative stress or excess FFA as well as other factors [8, 143–145] (Figure 3). Under oxidative stress conditions, superoxide radical reacts with NO resulting in the formation of ONOO• and decreased NO bioavailability [146]. The generation of free radicals and activated endothelial cells starts the complex pathogenic events [147], which attract the circulating macrophages and internalize modified lipoproteins to become foam cells [148]; multiple cytokines and growth factors detailed by endothelial cells attract the adjacent smooth muscle cells to induce proliferation and production of the extracellular matrix within the inner layer of vessels which ultimately results in generation of fibromuscular plaque [149].

Free radicals and ROS have a significant contribution in the pathogenesis of ED and CVD. The body cells and tissues are in continuous danger from free radicals and ROS which are generated during the normal process of metabolism. Thus, antioxidants can play a central role in boosting the cellular capacity against ROS-induced injury. The antioxidant activity of flavonoids is well-documented, and they protect cells from the lethal free radicals and ROS [150].

Normal arterial pressure is necessary for the healthy activity of the vasculature and normal blood flow. Citrus fruit flavonoids act as vasorelaxants and maintain vasculature tone throughout the body [150]. The vasorelaxant activity of citrus flavonoids also protects arterial intima from ED and from other diseases including metabolic syndrome [151].

A study conducted on spontaneous hypertensive rats (SHR) showed that a continuous 8-week duration of hesperidin intake can significantly reduce blood pressure, oxidative stress, ED, and cardiac and vascular hypertrophies. Moreover, G-hesperidin (alpha glucosyl hesperidin) intake showed an ability to increase acetylcholine-induced endothelium-dependent vasodilation among SHRs. The same study showed that the intake of G-hesperidin did not affect eNOS gene expression and was not responsible for the increased NO production [152]. In another study, where SHRs were treated with hesperidin, the results showed a dose-dependent inverse relation with ED and systolic blood pressure [153]. In a diabetic rodent model, the use of hesperidin resulted in hypoglycemia and reduced circulating FFA, triglycerides, and total cholesterol [154]. Human patients diagnosed with hyperlipidemia and treated with G-hesperidin showed lower circulating triglyceride levels [155, 156].

Naringin and naringenin are known as sturdy free radical scavengers and help in the prevention of lipid peroxidation. In an *in vitro* study, superoxide and hydroxyl radicals were scavenged by these flavonoids [157]. Naringin has the ability to inhibit the activity of XO, an indigenous source of superoxide anions in eukaryotic cells [158]. A study conducted on diabetic rats, where the rats were supplemented with naringin, showed improved and enhanced activity of antioxidant enzymes including SOD, catalase, and GPx [159]. Another study conducted by Jeon et al. [160] on cholesterol-fed rabbits showed that naringin supplementation can increase the activity of antioxidant enzymes; however, the TBARS concentration remained unchanged.

### 4.2. Citrus Flavonoids and Atherosclerosis

Citrus flavonoids have gained special attention among others, because of their unique and enhanced therapeutic properties against different chronic diseases, particularly atherosclerosis [161, 162]. Flavonoids have very specific antioxidant properties and can protect cells against oxidative damage [162]. A study carried out by Gorinstein et al. showed that the intake of citrus fruit reduces the plasma level of triglycerides in CVD patients [163]. Another recent study on the daily intake of glucosyl hesperidin (500 mg/day for 6 or 24 weeks) showed significantly reduced triglycerides in both hyperlipidemia and hypertriglyceridemia subjects [155, 156]. A study in hypercholesterolemia patients revealed that the intake of naringin (400 mg/day for 8 weeks) can cause 17% reduction in LDL-C and apoB level in plasma [164]. 0.05% naringenin and 0.1% naringin were given to high cholesterol-fed rabbits, and the results showed a reduction in aortic fatty streaks [165].

Different cell model studies were performed for the evaluation of citrus flavonoids. A previous study on HepG2 furnished evidence that both naringenin and hesperetin reduced apoB100 accumulation over the media for four hours [80]. Different studies on HepG2 cells showed that naringenin inhibits cholesterol acyltransferase and microsomal triglyceride transfer proteins which limits cholesteryl ester and triglyceride availability for the formation of lipoprotein [166, 167].

A study was conducted on C57BL/6 mice, where HFD containing 0.5% lemon peel polyphenols such as eriodictyol and hesperidin, demonstrated significantly reduced plasma triglyceride and hepatic lipid levels [168]. Peel extract of *Citrus reticulata* administered into the *db/db* mice caused a decrease in liver fat and reduction in plasma lipids [169]. Wistar rats fed a high-cholesterol diet along with the administration of naringenin (50 mg/kg) for 90 days showed marked reduction in plasma lipids, hepatic lipids, and fibrosis associated with reduced matrix metalloproteinase gene expression and markers of macrophage infiltration [170].

A clinical study of subjects with hypercholesterolemia (cholesterol >230 mg/dl), who received 270 mg of citrus flavonoid and 30 mg of tocotrienols daily for the period of four weeks, revealed significant reductions in total plasma cholesterol (20-30%), LDL (19-27%), and TG (24-34%) [171]. Intake of orange juice (480 ml/day for 1 year) reduced the concentration of total cholesterol, LDL cholesterol, and apoB in patients with mild hypercholesterolemia [172]. Glucosyl hesperidin (500 mg/day for 24 weeks) supplemented to hypertriglyceridemic patients significantly reduced plasma triglyceride and apoB [155]. A larger study conducted on Japanese subjects (10,623 participants: 4,147 male and 6,476 female) using citrus fruit intake (6-7 times/week) demonstrated an inverse association for CVD, specifically ischemic stroke [173].

Rabbits fed cholesterol and a daily intake of 500 mg/kg naringin supplementation showed reduced vascular fatty streak arrangement and macrophage infiltration in vascular walls. In the same study, hypercholesterolemic rabbits treated with naringin showed antiatherogenic activity by inhibiting ICAM-1 expression in endothelial cells [174]. In another study, rabbits with high plasma cholesterol were treated with naringin and naringenin and both showed antiatherogenic effects by downregulating the expression of aortic VCAM-1 and MCP-1 [165]. Increased production of apoB containing lipoproteins is a characteristic feature of dyslipidemia along with insulin resistance [175]. When wild-type mice were supplemented with elevated levels of fat in their diet and naringin, the results showed significant antiatherogenic effects [176]. Naringin can also inhibit apoB100 secretion by stimulating the signaling cascade in HepG2 cells [177]. *Ldlr-/-^−^* mice fed with western diet and supplemented with 3% of diet with naringenin (w/w) showed a reduction in infiltration of MOMA-2-positive lesions and collagen deposition, which suggests the antiatherogenic activity [178].

Atherosclerosis is a very common disease worldwide, and multiple factors, including hypertension, diabetes, and high plasma cholesterol level, can accelerate its onset. Several medical therapies are available for the treatment of atherosclerosis, but they may have side effects. However, nutritional therapy and balanced diet have gained significant importance in recent years for the treatment of atherosclerosis and other cardiovascular diseases. The use of citrus fruits in daily diet not only provides valuable vitamins and nutrients to the body but also can enhance the metabolism of the body. The flavonoids present in citrus fruits have antioxidant, hypolipidemic, and antidiabetic activities and demonstrate a significant role in the control of free radicals. Therefore, citrus flavonoids could be of significant value as a treatment regime for counteracting atherosclerosis. However, clinical studies for the proper evaluation of citrus flavonoids metabolic activity are needed.

## 5. Citrus Flavonoids and Modulation of Platelet Function

Thrombocytes or platelets play a crucial role in hemostasis and wound healing. However, excessive activation of thrombocytes is associated with many disorders, including DM and hypertension. In addition, platelet dysfunction participates in the pathogenesis and progression of thrombosis and CVD [179]. Flavonoids possess multiple therapeutic benefits against cancer, neurodegenerative disorders, and CVD. Given their antihyperlipidemic effects and their regulatory role in lipid metabolism, flavonoids can reduce cell adhesion and improve the function of vascular endothelium [179–181]. Therefore, flavonoids have been proposed as novel candidates for the development of therapeutic agents counteracting several disease conditions associated with thrombotic events [182, 183].

Epidemiological reports have pointed to the inverse relationship between platelet activity and the consumption of citrus flavonoids. Hence, citrus flavonoids can play a protective role against the pathogenesis and progression of CVD [179, 183–186]. Flavonoids are capable of inhibiting platelet function and hence might be of value as antithrombotic agents [183, 186, 187].

The exact mechanisms underlying the antiplatelet activity of citrus flavonoids are not fully elucidated. Studies have demonstrated different mechanisms describing the effect of flavonoids on platelet function. Inhibition of the arachidonic acid-based pathway has been postulated as the primary effect of flavonoids in platelets [179, 180]. Other mechanisms such as mobilization of intracellular Ca^2+^, attenuation of agonist-induced GPIIb/IIIa receptor activation, and activation of phospholipases and MAPK have been proposed for the reduced platelet activity by flavonoids [182]. However, Ravishankar et al. have recently reported that the underlying mechanism depends specifically on the flavonoid structure and the included functional groups [183]. The antiplatelet activity of citrus flavonoids naringin and naringenin as well as other compounds, such as, coumarin, esculetin, and fraxetin has been tested [186]. Naringin and naringenin showed a more potent ability to bind to and inhibit GPIIb/IIIa receptors which have a role in platelet activation by acting as receptors for fibrinogen and von Willebrand factor [186]. Naringenin improved NTPDase activities in platelets in hypercholesterolemic diet-fed rats [188]. In addition, citrus flavonoids may have an impact on the circulating levels of fibrinogen, factor (F)VII, and plasminogen [184, 187, 189].

The citrus flavonoid tangeretin has also shown antiplatelet activity mediated via inhibition of intracellular calcium mobilization, GPIIb/IIIa receptor signaling, granule secretion, platelet adhesion, and thrombus formation [190]. The impact of tangeretin on platelets has been attributed to inhibition of PI3K signaling and increased cGMP levels in platelets [190].

Nobiletin has also been investigated for its antiplatelet activity. Both *in vitro* and *in vivo* experimental studies demonstrated the ability of nobiletin to suppress platelet aggregation, calcium mobilization, granule secretion, and thrombosis. In C57BL/6 mice, nobiletin reduced Akt phosphorylation, increased cGMP, suppressed phospholipase PLC*γ*2 and vasodilator-stimulated phosphoprotein phosphorylation, and extended bleeding time [187]. In addition to the previously mentioned effects, nobiletin suppressed the phosphorylation of Akt, MAPK, and PLC*γ*2 as well as ROS levels in collagen-activated human platelets [185]. Furthermore, incubation of human platelets with nobiletin resulted in increased phosphorylation of vasodilator-stimulated phosphoprotein, a substrate of cAMP and cGMP-regulated protein kinases [191].

## 6. Concluding Remarks

**Figure 4:**
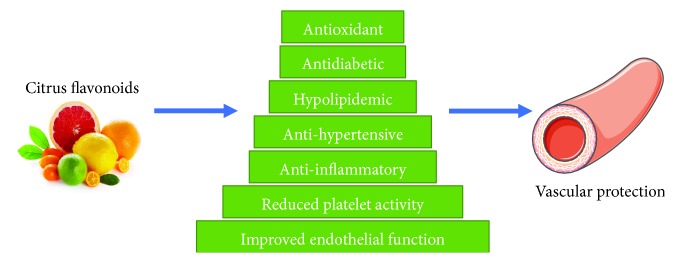
Citrus flavonoids confer vascular protection via their antioxidant, antidiabetic, anti-inflammatory and other biological activities.

- The available data suggests that citrus flavonoids are likely to confer protection against CVD. The ability of citrus flavonoids to reduce oxidative stress, hyperlipidemia, and inflammation and to improve endothelial function, arterial blood pressure, and lipid metabolism may be responsible for their therapeutic role against atherosclerosis and CVD (Figure 4).
- *In vitro* and *in vivo* studies indicate that citrus flavonoids protect against ROS-induced cell injury, reduce obesity and adipose tissue inflammation, and improve platelet function. Citrus flavonoids modulate several signaling pathways controlling inflammation and other processes such as NF-*κ*B
- Studies in experimental diabetes models demonstrate the efficacy of citrus flavonoids to improve glucose tolerance, increase insulin secretion and sensitivity, decrease insulin resistance, reduce hepatic glucose output and intestinal glucose absorption, enhance peripheral glucose uptake, suppress inflammation, and modulate activity of enzymes and transporters involved in glucose and lipid metabolism
- Citrus flavonoids modulate different signaling pathways involved in adiposity and adipocyte differentiation and hence could be of significant value for the development of antiobesity agents
- Given the tremendous increase in the number of diabetic patients in the world, there is a greater concern for the development of harmless, efficient, and cost-effective antidiabetic medicine. Therefore, further studies and clinical trials to assess the efficacy and to explore the underlying citrus flavonoids mechanism(s) of action are recommended in both healthy subjects and patients. The results of these studies might open new avenues of research in the development of novel therapeutic agents
